# When peer review never happens: who decides what science deserves to be reviewed?

**DOI:** 10.2340/aos.v85.47059

**Published:** 2026-10-26

**Authors:** Pekka Vallittu

**Affiliations:** Department of Biomaterials Science, Institute of Dentistry, University of Turku, Finland

One of the fundamental principles of science is that scientific claims should be evaluated objectively and transparently. Peer review is a cornerstone of this principle. Independent experts, selected by editors and drawn from the scientific community, assess the quality, validity, originality, and significance of submitted research. No system is perfect, but peer review provides an essential safeguard against scientific judgement being determined by a single individual.

Today, however, this principle is under increasing pressure. The number of scientific manuscripts submitted to journals has increased dramatically, in *Acta Odontologica Scandinavica* and other journals too [1]. At the same time, editors face growing difficulties in finding qualified reviewers who are willing and able to evaluate manuscripts. The consequence is increasingly extensive editorial triage, particularly at major journals receiving very large numbers of submissions. Manuscripts may be rejected at the editorial stage, often within days, without being evaluated by independent external reviewers.

There is, of course, a legitimate place for editorial rejection. A manuscript that is clearly outside the scope of a journal, does not meet basic methodological requirements, or is evidently unsuitable for its readership should not consume the time of reviewers. Editors have a responsibility to protect the limited resources of the scientific community.

But we should ask a more fundamental question: when a very large proportion of submitted research never reaches independent peer review, can we still claim that the scientific evaluation process is sufficiently transparent and objective?

A desk rejection is not peer review. It is an editorial prediction that a manuscript does not deserve further scientific evaluation by the journal. This distinction matters. Editors are highly experienced scientists, but they cannot necessarily determine from an initial reading whether an unconventional hypothesis is correct, whether an unexpected finding is scientifically important, or whether a technically sound study may have implications beyond its immediate field. These are precisely the questions that independent peer review is intended to address.

But the responsibility does not lie with editors alone. The underlying causes are structural. Universities and researchers are under enormous pressure to publish ever more. Research funding, institutional evaluations, career advancement, and international rankings are increasingly linked to publication output and perceived scientific impact. Governments, funding agencies, and universities have created incentives that continuously increase the number of manuscripts entering an already overloaded publication system. The result is a paradox: we demand more research, more publications, and faster dissemination of knowledge, while simultaneously creating a system that lacks the capacity to evaluate this research properly.

This is not merely an editorial problem; it is a challenge facing the entire research ecosystem. Funding agencies, ministries, universities, and research institutions must therefore recognize their role in creating the conditions under which peer review is becoming increasingly difficult to sustain. If society expects science to be objective, transparent, and trustworthy, it must also ensure a research and publishing environment in which independent scientific evaluation remains feasible.

Researchers themselves also have a responsibility to contribute actively to the peer-review process. Peer review is a collective responsibility and an essential service to the scientific community, not merely an obligation placed on journal editors. Those who benefit from the work of reviewers should also be willing to contribute their own expertise to the critical evaluation of research submitted by others. In this context, special recognition and sincere thanks are due to the dedicated researchers who serve on journal Editorial Boards and to all those who contribute their time and expertise to reviewing manuscripts, often without compensation and alongside their own demanding research and academic responsibilities. Their voluntary and largely invisible work is indispensable to maintaining the quality, integrity, and credibility of scientific publishing. Without their commitment, the peer-review system cannot function.

Ultimately, the question is not whether editors should be allowed to reject manuscripts without peer review. They must retain this right. The question is how far this practice can expand before we fundamentally change what we mean by peer-reviewed science. This is an issue that editors, reviewers, researchers, universities, funding agencies, and policymakers should all be willing to address.
